# “RéaNet”, the Internet utilization among surrogates of critically ill patients with sepsis

**DOI:** 10.1371/journal.pone.0174292

**Published:** 2017-03-30

**Authors:** Yên-Lan Nguyen, Raphaël Porcher, Laurent Argaud, Lise Piquilloud, Christophe Guitton, Fabienne Tamion, Sami Hraiech, Jean-Paul Mira, on behalf of “RéaNet” collaboration group

**Affiliations:** 1 Medical intensive care unit, Cochin hospital, AP-HP, Paris Descartes University, Paris, France; 2 Anesthesiology and surgical critical care medicine department, Cochin academic hospital, APHP, Paris Descartes University, Paris, France; 3 Clinical epidemiology center, INSERM U1153, Hôtel-Dieu academic hospital, APHP, Paris Descartes University, Paris, France; 4 Medical intensive care unit, Hospices Civils de Lyon, Edouard Herriot academic hospital, Lyon, France; 5 Medical intensive care unit, Angers academic hospital, Angers university, Angers, France; 6 Medical intensive care unit, Nantes academic hospital, Nantes university, Nantes, France; 7 Medical intensive care unit, Rouen academic hospital, Rouen university, Rouen, France; 8 Medical intensive care unit, North hospital Marseille, AP-HM, Marseille, France; Azienda Ospedaliero Universitaria Careggi, ITALY

## Abstract

**Context:**

Health-related Internet utilization is common but its use by proxies of critically ill patients is unknown. Our objective was to describe the prevalence and the Internet utilization characteristics among surrogates of critically ill septic patients. We conducted a prospective observational study in French ICUs. Three survey instruments were used to describe ICU organization regarding information delivery, patients and surrogates characteristics.

**Results:**

169 surrogates of 146 septic patients hospitalized in 19 ICUs were included. One sixth of ICUs (n = 3, 16%) had their own website. Majority of patients were males (n = 100, 68%), aged 64±1 years old, with a SAPS2 score at 53±17 and required vasopressors (n = 117, 83%), mechanical ventilation (n = 116, 82%). More than one quarter required renal replacement therapy (n = 36, 26%). Majority of surrogates were female, in their fifties. Only one in five knew the word sepsis (n = 27, 16%). Majority of proxies internet users (n = 77; 55%) search on the internet about sepsis. The main motivation was curiosity. Majority of surrogates found the information online reliable, suitable for request and concordant. Prior use of health-related Internet (OR = 20.7 [4.30–100.1]), the presence of a nursing staff during family-physician meetings (OR = 3.33 [1.17–9.53]), a younger patient age (OR = 1.32 [1.01–1.72]) and renal replacement therapy requirement (OR = 2.58 [1.06–6.26]) were associated with health-related Internet use. Neither satisfaction with medical care or information provision, neither presence of anxiety-depression symptoms, were associated with health-related Internet use. Majority of surrogates (N = 76 (52%)) would have like receiving a list of selected websites on sepsis.

**Conclusions:**

Majority of proxies of critically ill patients with sepsis use Internet to learn more about sepsis. Internet utilization is independent of satisfaction with global ICU care, perceived quality of information delivery by doctors or the existence of anxiety-depression symptoms during ICU stay. The delivery of a list of recommended web sites on sepsis would have been appreciated.

## Introduction

During the last decade, the Internet has become a major source of educational materials for patients and health-care workers. Recent population-based surveys suggest that the majority of adults in developed countries surf the Internet to obtain medical information [1–2]. The main advantages of the Internet as a source of information are that it offers a large variety of health-related websites (from online health discussion forums and medical online support groups to the national library of medicine) and is accessible 24/7. Its main drawbacks are quality heterogeneity and the quasi-inexistent regulation or control of these websites. Diverse e-programs are currently tested among chronically ill patients in order to enhance adequate self-management [3–4]. The situation is different in critical care, as patients do not usually choose to be hospitalized in intensive care unit (ICU) and when they are hospitalized, they are often unable to use the Internet.

The use of the Internet by family members and friends of critically ill patients in life threatening conditions like septic shock is unknown. The stress and anxiety induced by the unplanned admission and the hostility of the ICU environment may lead proxies to search for health information online like testimonies. The discovery that an infection, although treated with the right antibiotics, may lead to multiple organ failure and impair survival, may be frightening to proxies, most of whom may never have heard of the term “sepsis” before. This may therefore lead to Internet searches on “sepsis”[5]. Recent data suggests that the majority of patients do not share online findings with their physicians [1,6]. The main underlying reasons are; being skeptical about physician reactions, the resistance or discouragement of physicians and the fear of embarrassment [7]. On the other hand, the patient-physician relationship is often positively affected when patients have the opportunity to share and discuss their online health information with their physicians [7].

A better understanding of critically ill patients proxies’ information needs may help physicians to adjust their medical information delivery, to encourage them to discuss with proxies of their Internet searches and to avoid reactions perceived as negative by proxies.

We hypothesized that the lack of understanding amongst family members of critically ill patients with sepsis should lead them to seek supplementary information online. The aim of our study is to describe the health-related Internet use characteristics among surrogates of critically ill patients with severe sepsis or septic shock in French ICUs.

## Methods

This study was approved by the ethics committee of the French Intensive Care Society (FICS/SRLF).

### Setting and participants

This longitudinal prospective study was conducted in 19 ICUs in France in 2013. The ICUs were selected on the basis of homogeneous practices regarding family-centered and sepsis care and spread out throughout France.

Patients were selected if they had severe sepsis or septic shock (in accordance with the 2001 SCCM/ESICM/ACCP/ATS/SIS International Sepsis Definitions Conference), if they were unable to participate in decision-making and if they had at least one proxy who had visited them at bedside [8]. In each center, a local investigator identified relatives of selected patients to participate between day 2 and 5. All relatives fluent in French and identified as decision making surrogates were invited to participate to our study. Surrogates were able to decline the invitation to participate. Each participating ICU included consecutive patients during a two months period.

### Survey instruments and procedures for data collection

#### Questionnaire on ICU organization and management (S1 File)

Data on academic status, number of beds, annual admissions, nurse-to-patients ratio, presence of a social worker or psychologist in the unit, were collected. Visiting hours policy, medical information delivery (use of a written protocol to interact with families, frequency of participation of junior doctors and nursing staff to the information meeting (Likert scales)), family information leaflet availability (following the recommendations of the French Intensive Care Society_SRLF), existing own ICU website, Internet access to patients and visitors availability were also collected.

#### Questionnaire on critically ill patient (S1 File)

The instrument survey was completed by the local investigator. Data included: demographic data (age, gender, MacCabe score), sepsis description (sepsis origin, SAPS2, organ supply) and patient related outcomes (ICU length of stay, decision to withdrawal/withhold life sustaining therapies, ICU mortality). All data were collected anonymously.

#### Questionnaire on Internet use characteristics, satisfaction, anxiety-depression evaluation (S1 File)

The lack of gold standard leads us to use in our questionnaire several questions from a national survey conducted by the French Institute of polls (IPSOS) [1]. We tested our questionnaire among 10 persons who were not physicians to limit information bias.

The survey instrument was completed by surrogates of patients, between day 2 and day 5 after ICU admission. We chose this delay to allow time to relatives to meet the senior physician in charge of the patient and to cope with the stress and anxiety induced by the hospitalization. A physician (local investigator), independent of patient care, delivered the questionnaire to surrogates who were identified as relatives that previously received medical information. Data was collected anonymously. Demographic data, Internet use description (general use and health-related), prior visit to an ICU, reception of an information family leaflet, knowledge of the term “sepsis”, knowledge of existing health web sites certification, willingness to obtain a list of websites on sepsis, global satisfaction with ICU care (Likert scale), satisfaction with medical information delivery (using the 7 items on communication of the Family Satisfaction in the ICU 24 scale (FS ICU 24)), anxiety and depression (using the Hospital Anxiety and Depression scale (HAD)) were collected [9]. HAD scale is a 14 items questionnaire subdivided in two subscales (HAD_A for anxiety and HAD_D for depression). Each item is coded 0 to 3, which gives a score varying between 0 and 21 for each subscale. A score of less than 8 means the lack of significant anxious or depressive symptom.

All questionnaires were sent to primary investigators (YLN, RP, and JPM).

### Statistical analyses (S1 File)

Associations of ICU, patient and responder's characteristics with Internet use were analyzed using logistic regression models (three-stage procedure). First, basic characteristics of the responder were analyzed in a multivariable model to determine a set of potential confounders. Then a potential clustering effect on the center was investigated using a mixed effects logistic regression model, with a random center effect. The standard deviation of the center effect relative to the effect of adjustment variables was taken as a measure of clustering, and tested using a permutation test. In the third step, other variables related to the ICU, the patient or the responder where analyzed [10].

Analyses were performed using the R statistical software version 3.0.2 [11].

## Results

### Setting (S1 and S2 Tables)

19 ICUs participated in the study (5 ICUs refused to participate and one did not answer to our invitation). The majority of ICUs included were located in academic hospitals (N = 17 (89%)) with a median number of beds of 16 (range 14–20). The median annual number of total admissions was 900 (660–1164). The median annual number of admissions with sepsis was 100 (100–142). The median number of senior physicians and residents was respectively 8 (7–9) and 6 (4–8). The average mortality rate was 21±7%.

Among the 19 ICUs, 3 ICUs (16%) had their own website and none of the participating ICUs offered Internet access to patients or visitors. All ICUs had a waiting room. Majority of ICUs (N = 17 (89%)) had a room used only for family information and had a family information leaflet available. A social worker was available in 17 ICUs (89%). A psychologist was available for families in more than half of the ICUs (N = 10 (53%)). Nearly one third (N = 6 (32%)) had a 24/7 visiting hours policy. A minority of ICUs (N = 2 (11%)) had a written protocol for interacting with families. Family information was delivered by residents very occasionally in 74% of ICUs. In half of the ICUs (N = 9 (47%)), the nursing staff was present during family information meetings.

### Patients (S3 Table)

146 patients were included in our study. The majority of them were male (N = 100 (68%)), average age = 64±16. Half of the patients had a MacCabe category 1 (N = 75 (51%)). The source of sepsis origin was identified accordingly: pulmonary (N = 70 (48%)), abdominal (N = 25 (17%)), urinary (N = 16 (11%)), skin and soft tissues (N = 13 (9%)) and others (N = 22 (15%)). The average SAPS2 score was 53±17. The majority of patients received vasopressors (N = 117 (83%)) and invasive mechanical ventilation (N = 116 (82%)). One fourth of patients (N = 36 (26%)) received renal replacement therapy. Around one fifth of patients had decisions to withdrawal or withhold life-sustaining therapies during their ICU stay (N = 30 (21%)). The median ICU length of stay was 11 (7;18) days and around one fourth of the patients died (N = 35 (24%))

### Surrogates

In total, 169 surrogates answered the questionnaire. The majority were female (N = 119 (71%)), in their fifties (51±16 years). Relationships with the patient were partners (N = 66 (39%)), children (N = 57 (33%)), parents (N = 15 (9%)), siblings (N = 12 (7%)) and others (N = 18 (11%)). Half had an academic degree (N = 82 (50%)). 80 (48%) respondents had already been exposed to the ICU experience previously. The majority (N = 108 (65%) were “very satisfied” with global ICU care. The anxiety degree amongst surrogates was normal-35 (22%), (HAD-A score [0–7]), moderate-31 (20%), (HAD-A score [8–10]), mild-48 (30%) (HAD-A score [11–14]) and severe-44 (28%) (HAD-A score [15–21]). More than half (N = 90 (59%)) of surrogates had a normal depression scale (HAD-D score [0–7]). Only a minority (N = 27(16%)) of surrogates knew the term “sepsis”.

#### Surrogates and satisfaction of communication with doctors

All surrogates had received medical information on their proxies. Less than half (N = 65 (41%)) received a family information leaflet. Majority (N = 137 (84%)) perceived the frequency of communication with doctors “very frequent” or “excellent”. Majority perceived as “excellent” or “very good” the ease of getting information (N = 141 (85%)), the understanding of information (N = 136 (83%)), the honesty of information (N = 135 (82%)) or the completeness of information (N = 130 (78%)) and the consistency of information (N = 118 (71%)).

#### Surrogates and the Internet use characteristics (S4 and S5 Tables)

Majority of surrogates (N = 139 (83%)) used the Internet regularly for general purpose. Among them, 115 (83%) went online in search for health information. The hospitalization of their proxy in critical care leads to health-related Internet use among 77 surrogates (55% (N = 76) of the Internet users and 3% (N = 1) of non-users). The Internet users connected at home (N = 125 (79%)), with their smartphone (N = 49 (31%)) or at work (N = 26 (16%)). Only a few knew about the certification of health web sites called “Health on the Net” (N = 12 (8%)). More than half of surrogates (N = 76 (52%)) would have liked to receive a list of selected websites on sepsis.

#### The Internet users characteristics (S6 Table)

**Fig 1:**
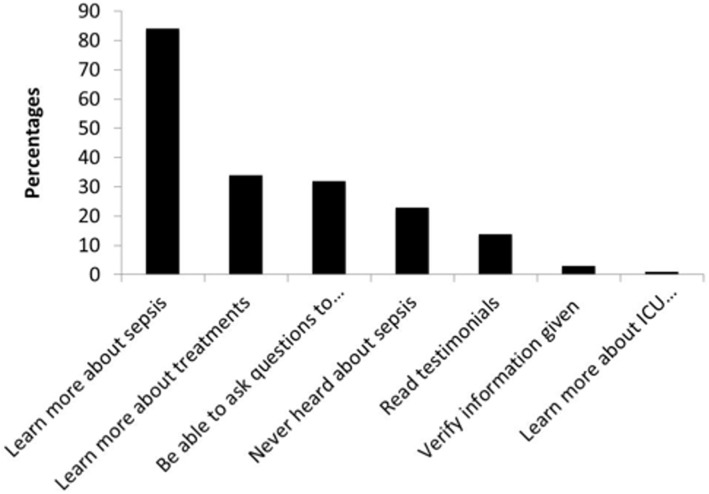
Motivations to use the Internet to search for health-information.

The single surrogate individual factor associated with the Internet use was the previous use of the Internet for health information (OR = 20.7 (4.30–100.1)). Age, gender or education level, were not associated with the Internet use. The main motivations to seek health information on their proxy were to “learn more about sepsis” (N = 65 (84%)), “learn more about treatments” (N = 26 (34%)), “be able to ask questions to the physicians” (N = 25 (32%)) or “because they had never heard about sepsis before” (N = 18 (23%)) (Fig 1).

**Fig 2:**
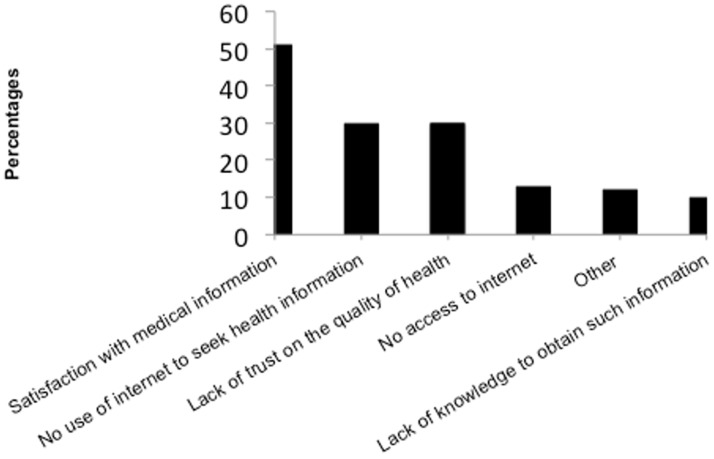
Motivations to not to use Internet to search for health-information.

One third (N = 24 (33%)) informed their doctors that they had consulted Internet. The main reasons of not telling the doctor about Internet searches were “curiosity and not necessary to share it with the doctors” (N = 36 (72%)), or “the willingness to do comparisons” (N = 5 (10%)). The main reasons for those who did not use the Internet, were “satisfaction with medical information” (N = 46 (51%)), “no access to the Internet” (N = 39 (43%)) and “no trusting the quality of health information online” (N = 27 (30%)) (Fig 2).

**Fig 3:**
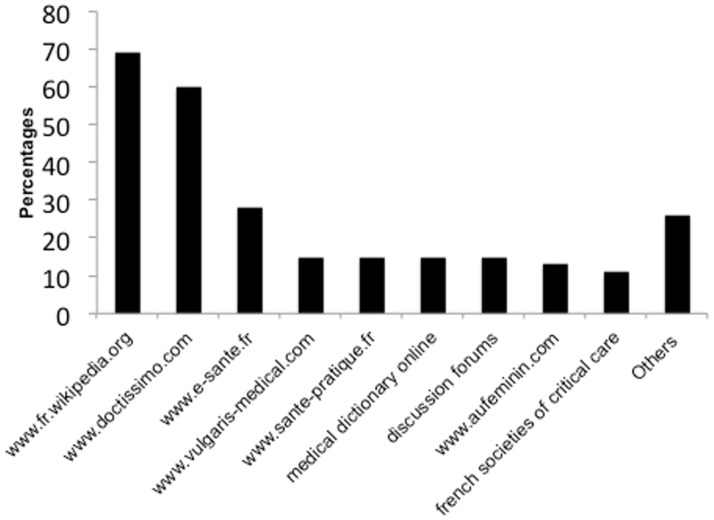
List of websites most frequently used.

The majority used an Internet search engine on first line (N = 62; 79%). Websites most frequently cited were www.fr.wikipedia.org (online encyclopedia)(N = 52 (69%)), www.doctissimo.com (health website)((N = 45 (60%)), www.e-sante.fr (health website) (N = 21 (28%)), www.vulgaris-medical.com (health website) (N = 11 (15%)), www.sante-pratique.fr (health website) (N = 11 (15%)), medical dictionary online (N = 11 (15%)) and discussion forums (N = 11 (15%)) (Fig 3).

The majority of Internet users agree that they found “reliable information” (N = 52 (69%)), “appropriate information” (N = 45 (60%)), “concordant information”(N = 46 (62%)) on the Internet. Half (N = 37) of Internet users assess as “good” the quality information found on the Internet (Table 1).

**Table 1:** Evaluation of health-information found online.

Information characteristics	Strongly agree	Agree	Disagree	Strongly disagree	Undecided
Reliable (%)	9	69	1	0	20
Suitable for the request (%)	13	60	13	1	12
Concordant (%)	20	62	1	1	0

### ICU-related factors with Internet use (Table 2)

**Table 2:** ICU related factors associated with health-related Internet utilization (analyses adjusted on usual health-related Internet use).

Factors	OR [95% CI]	p
ICU website	1.13 [0.24–5.32]	0.88
Room dedicated to information	1.05 [0.38–2.90]	0.93
Information leaflet	0.79 [0.25–2.51]	0.69
24/7 visiting hours	0.85 [0.40–1.80]	0.66
Written procedure of information	1.82 [0.79–4.17]	0.16
Nursing staff very often present during meetings with families	3.33 [1.17–9.53]	0.025

ICU organization characteristics, an information leaflet delivery, the availability of a social worker or psychologist, the existence of a written procedure of information delivery, a 24/7 visiting hour’s policy did not influence Internet use among surrogates. The presence of a nursing staff member during family-physician meetings was associated with more frequent Internet use (OR = 3.33 [1.17–9.53]).

### Patient-related factors with Internet use (Table 3)

**Table 3:** Patient-related factors associated with health-related Internet utilization (analyses adjusted on usual health-related Internet use).

Factors	OR [95% CI]	p
Age (/10yrs)	1.32 [1.01–1.72]	0.041
SAPSII (/10units)	0.88 [0.71;1.08]	0.21
Vasopressors	0.94 [0.32;2.77]	0.92
Mechanical ventilation	2.23 [0.84;5.95]	0.11
Renal replacement therapy	2.58 [1.06;6.26]	0.036
Withdrawal or withholding therapies decisions	0.48 [0.20;1.11]	0.085

A younger age (OR = 1.32 [1.01–1.72]) and renal replacement therapy requirement (OR = 2.58 (1.06–6.26)] were associated with health-related Internet use. Patient severity (OR = 0.88 [0.71–1.08]), comorbidities (OR = 0.65 [0.25–1.67]), vasopressors (OR = 0.94 [0.32–2.77]), or mechanical ventilation (OR = 2.23 [0.84–5.95]) requirement, decisions to forgo life-sustaining therapies (OR = 0.48 [0.20–1.11]), were not associated with Internet use.

### Surrogate-related factors with Internet use (Table 4)

**Table 4:** Surrogate-related factors associated with health-related Internet utilization (analyses adjusted on usual health-related Internet use).

Factors	OR [95% CI]	p
Lack of satisfaction with ICU care	1.39 [0.69–2.77]	0.36
Lack of satisfaction with medical information provision	0.82 [0.39–1.75]	0.61
Presence of anxiety symptoms	1.05 [0.97–1.13]	0.26
Presence of depression symptoms	1.03 [0.95–1.12]	0.44

The lack of satisfaction with ICU care (OR = 1.39 [0.69–25.77]) or with medical information provision (OR = 0.82 [0.39–1.75]) and the presence of anxiety (OR = 1.05 [0.97–1.13]) or depression symptoms (OR = 1.03 [0.95–1.12]), were not associated with Internet use.

## Discussion and conclusions

To our knowledge, this is the first study on health-related Internet utilization among surrogates of critically ill patients with sepsis. We found that such a habit is frequent and independent of socio-demographic characteristics or satisfaction with care. The main reasons are curiosity and a tool to enhance discussions with physicians. Predictive factors of Internet utilization are prior health-related Internet use, a young patient age, renal replacement therapy requirement and the presence of nursing staff during family-physician meetings. We also found that the term “sepsis” remains relatively unknown by the general public and that half of patient surrogates would have liked to have received a list of recommended websites on sepsis.

First, we found that health-related Internet utilization by surrogates of critically ill patients is frequent and is not associated with socio-economic patterns. Our results confirm those of a French pilot study showing in a general ICU population that 45% of family members use the Internet for health-related searches [12]. In comparison to the study of Dhillon et al. conducted among parents of Neonatal ICU patients, published in 2003, we did not find that a younger age or a high education level were associated with Internet use [13]. Among the potential underlying reasons there are: Internet access democratization (via smartphones, tablets, at work or Internet coffee shops) and an increasing number of health websites and discussion forums during the last decade.

Secondly, we found that health-related Internet utilization by surrogates of critically ill patients is mainly driven by curiosity and is not associated with anxiety symptoms existence or with a lack of satisfaction with care. Similarly to the results of a French national survey conducted on general population, we found that curiosity (“to learn more about the diseases and its treatments”) is one of the main reasons that drives health-related Internet utilization [1]. Such curiosity, may be related to the wish of “being ready” in case of having to be part of the decision making process (recently legislated in France).

The lack of an existing association between Internet utilization and anxiety symptoms existence might be explained by the different perceptions on stress, of medical information found online. On one hand, it may be perceived as a strategy for coping with stress. On the other hand, it may be perceived as a potential anxiety factor (fear to read only “extreme” personal histories).

Unlike the results of Martinez et al., we did not find any association between Internet utilization and dissatisfaction with care [14]. This discrepancy in results may be related to the quality of communication. In our study, the majority of proxies perceived the frequency of communication with doctors being “very frequent” or “excellent”, as well as the understanding of information and proxies dissatisfied with care being very rare (<1%). Our high rates of satisfaction with communication are similar to those reported by Azoulay et al. [15]. On the other hand, in a large cohort of COPD patients, Martinez et al. found a higher percentage of patients dissatisfied with care (between 5–23%) and those who reported that “the doctor does not listen” or “insufficient time with doctor”, had a more frequent Internet use (respectively 3.14 [1.42, 6.95], 2.29 [1.02–5.13]) [14].

Thirdly, we found that a prior health-related Internet use, a young patient age, renal replacement therapy requirement and the presence of nursing staff during family-physician meetings are predictive factors for going online to search for medical information. Similarly to the pilot study of Bouju et al. we found that surrogates accustomed to search online for medical information, are prone to do it during the ICU stay of their proxy [12]. We suspect that such behavior may be related to the high satisfaction associated with the medical information found online. In our study, the majority of surrogates found the e-information reliable, suitable for the request and concordant with medical information. These results are discrepant with those of Dhillon AS, who found that only 10% of respondents report the e-information as being reliable [13]. Such difference may be related to the enhanced quality of health related websites during the last decade and to the creation of health web sites certification (Health on the Net).

The finding of “a young patient age” and “renal replacement therapy requirement” being two predictive factors of Internet utilization was unexpected. The occurrence of a life threatening condition is less frequent among young adults rather than with elderly and such situation often raises questions such as «why did that happen to her/him?». Similarly to patients with chronic kidney disease or the elderly, surrogates are often fearful of the multiple constraints of dialysis [16].

The more frequent use of Internet by surrogates when nursing staff participated in the family-physician meeting may be related to a greater curiosity raised by a better understanding of the situation. Indeed, the presence of a nurse during medical interview, may lead physicians to be more attentive to their language and to provide more comprehensive information. It is also likely that the nurse may participate actively to the conversation and clarify medical terms if necessary.

Lastly, despite sepsis being the leading cause of death in the ICU, the term“sepsis” and its life threatening condition seems relatively unknown (less than one surrogate in five knew it). Our results confirm the poor public awareness of the term“sepsis” described in two populations based surveys [17;5]. Despite several initiatives (Global Sepsis Alliance, Surviving Sepsis Campaign, “World Sepsis day”), sepsis remains a term relatively unknown in mass media, as illustrated in Google trends (large differences observed between the trends of the word sepsis versus pneumonia at the international level) [18–20].

Limitations of our study include those related to study design. Our selection criteria for participating ICUs and surrogates make it difficult to generalize our results to all critically ill patients. Despite the multicenter aspect of the study, answers to our questionnaire may have been different in other health care systems. The purpose of our study did not allow us to evaluate the impact of Internet utilization (to date a list with recommended websites on sepsis is lacking) on anxiety and depression symptoms at 3 months of surrogates of critically ill patients.

In the era of widespread use of the health-related Internet, intensivists, like other physicians should take in account the fact that majority of the families of critically ill patients seek medical information online. Our results should prone physicians to discuss with families about information found on line and to recommend them websites dedicated to sepsis vulgarization. Such behaviors may enhance trust and satisfaction in family-physician relationships. Further qualitative studies are needed to better understand patients and families expectations and especially the type of online information on “sepsis” they would like to share with hospital staff.

## Supporting information

S1 Dataset Base reanet janvier 2014 YLN. (XLSX)

S1 File Questionnaires on ICU organization and management, on critically ill patient, on Internet use characteristics, satisfaction, anxiety-depression evaluation and details on statistical Analyses. (DOCX)

S1 Table ICUs characteristics. (DOCX)

S2 Table Centers organization on medical information. (DOCX)

S3 Table Patients characteristics. (DOCX)

S4 Table Internet use characteristics. (DOCX)

S5 Table Internet users characteristics. (DOCX)

S6 Table Potential confounders of Internet utilization. (DOCX)
