# Supplementary material for: “RéaNet”, the Internet utilization among surrogates of critically ill patients with sepsis
Source: PLoS One. 2017 Mar 30;12(3):e0174292. doi: 10.1371/journal.pone.0174292 (PMC5373530; doi:10.1371/journal.pone.0174292)
Supplement: S1 File — (DOCX) [file pone.0174292.s002.docx]

**Supplementary file S1**

Questionnaire on ICU organization and management

Questionnaire on critically ill patient

Questionnaire on Internet use characteristics, satisfaction, anxiety-depression evaluation

Statistical Analyses

1. Questionnaire on ICU organization and management

**Nom :**

**Service :**

**Centre Hospitalier :**

1. **Nombre total de lits dans votre service :**

Nombre de lits dédiés « réanimation » :

Nombre de lits dédiés « soins continus » :

1. **Personnel Médical :**

Nombre de praticien(s) hospitalier(s) universitaire(s)

Nombre de maître(s) de conférence universitaire(s)

Nombre de praticien(s) hospitalier(s)

Nombre de praticien(s) hospitalier(s) contractuel(s)

Nombre de praticien(s) hospitalier(s) assistant(s)

Nombre de chef(s) de clinique assistant(s)

Nombre d’internes

Nombre d’externes

1. **Personnel para-médical :**

Ratio patients/IDE

Présence d’un(e) assistant(e) social(e)

Non

Oui, à temps partiel

Oui, à temps complet

Présence d’un psychologue

Non

Oui, à temps partiel

Oui, à temps complet

1. **Existe-t-il une salle d’information dédiée aux familles ?**

Non

Oui

1. **Existe-t-il une salle d’attente propre à votre service ?**

Non

Oui

**Si OUI à la question 7, y-a-t-il un accès à internet dans cette salle d’attente ?**

Non

Oui

1. **Remettez-vous un livret d’accueil aux proches sur le service de réanimation ?**

En systématique

A la demande

Jamais

1. **Que contient votre livret d’accueil ? (plusieurs réponses possibles)**

des informations sur l’organisation du service de réanimation

des informations sur le personnel

des informations sur les pathologies rencontrées dans le service

un lexique des termes médicaux fréquemment utilisés

des liens de site internet

Non applicable

1. **Quels sont les horaires de visites des familles dans votre service**?

- **Semaine : de ………h à ………h**
- **Week-end : de …….h à ………h**

1. **Existe-t-il dans votre service une procédure de soins (ou protocole) pour guider l’entretien avec les familles ?**

En systématique

A la demande

Jamais

1. **Quelle est la place des internes lors des entretiens avec les familles ?**

Les internes font très souvent les entretiens avec les familles seuls

Les internes font assez souvent les entretiens avec les familles seuls

Les internes font parfois les entretiens avec les familles seuls

Les internes ne font jamais les entretiens avec les familles seuls

Non applicable

1. **Quelle est la place du personnel para-médical (infirmier, aide-soignant, kinésithérapeute, assistant(e) social(e)) lors des entretiens avec les familles ?**

le personnel para-médical est très souvent présent

le personnel para-médical est assez souvent présent

le personnel para-médical est parfois présent

le personnel para-médical n’est jamais présent

1. **Existe-t-il un site internet « du service » ?**

Non

Oui

**Si OUI à la question 9, existe-t-il sur ce site, une ou plusieurs pages de vulgarisation médicale dédiée(s) aux familles ?**

Non

Oui

Je ne sais pas

1. **Existe-t-il dans votre service des consultations « post-réa » (des consultations de surveillance à distance du séjour en réanimation)?**

Non

Oui

1. **Nombre annuel d’admissions**
2. **Nombre annuel d’admissions pour sepsis sévère ou choc septique communautaire**
3. **Taux de mortalité annuel dans votre service**
4. **Selon vous, quel est approximativement le pourcentage de proches, consultant internet pour rechercher des informations médicales au sujet de l’hospitalisation en réanimation ?**
5. Questionnaire on critically ill patient
6. **Age :**
7. **Sexe :**

Masculin

Féminin

1. **Comorbidités (Mac Cabe) : le pronostic doit avoir été défini dans les 3 mois précédant l’entrée dans le service :**

Pas de maladie mortelle

Maladie mortelle à 5 ans :

- insuffisant cardiaque stade III NYHA
- insuffisant respiratoire sous 02 à domicile
- cancer non métastatique
- décompensation hémorragique de cirrhose

Maladie mortelle à 1an :

- insuffisant cardiaque stade IV NYHA
- insuffisant respiratoire déjà ventilé
- cancer métastatique
- hypertension portale

1. **Statut fonctionnel : Activité et suivi médical dans les 6 mois précédents l’admission**

Santé normale

Limitation modérée (présence d’un traitement chronique)

Limitation importante (handicap à l'effort, traitement anticancéreux, hémodialyse)

Patient grabataire, restriction majeure (hospitalisation long séjour, visite hebdomadaire d'un médecin, incapacité à 100 %)

1. **SAPS 2 :**
2. **Porte d’entrée du sepsis :**

pulmonaire

urinaire

digestif

cutané

autre, à préciser

1. **Suppléance d’organes :**

ventilation mécanique non invasive

ventilation mécanique invasive

catécholamines

épuration extra-rénale

autre, à préciser :

1. **Durée de séjour en réanimation (jours) :**
2. **Statut de limitation ou arrêt des thérapeutiques actives :**

Oui

Non

1. **Statut à la sortie du service de réanimation :**

Vivant

Décédé

1. Questionnaire on Internet use characteristics, satisfaction, anxiety-depression evaluation

*Une enquête récente de l’IPSOS révèle que près de* ***7 français sur 10 consultent internet pour rechercher des informations médicales****. Dès lors, il est possible que vous ayez consulté internet pour rechercher des informations sur la maladie de votre proche ou sur les motifs de son hospitalisation dans le service de réanimation.*

***Afin de mieux comprendre vos attentes et d’améliorer l’information médicale délivrée, nous effectuons une enquête pour évaluer l’utilisation d’internet comme source d’informations médicales chez les proches des personnes hospitalisées en réanimation pour une infection grave.***

*Vous êtes libre de participer à cette enquête. Il s’agit d’un questionnaire anonyme. Vos réponses ne seront pas transmises à l’équipe médicale prenant en charge votre proche.*

*La durée de complétion du questionnaire est d’environ 10min.*

*Cette étude a reçu un avis favorable de la commission d’éthique de la Société de réanimation de langue française le 28 Mars 2013.*

***Nous vous remercions très sincèrement par avance pour votre participation à cette étude.***

***Nous espérons que cette étude permettra d’améliorer la communication avec les proches des personnes hospitalisées dans les services de réanimation.***

*Cordialement,*

*Docteur YL Nguyen^1^et Professeur JP Mira^1^*

*^1^Service de réanimation, Centre Hospitalo-Universitaire Cochin, Université Paris Descartes, Paris*

**Acceptez-vous de participer à l’étude RéaNet?**

Oui

Non

***Avant votre venue dans le service de réanimation***

1. **Utilisiez-vous internet?** **(1 réponse)**

Oui

Non

1. **Quel(s) moyen(s) utilisez-vous pour vous connecter à internet ? (1 réponse)**

Ordinateur au domicile

Ordinateur sur le lieu de travail

Ordinateur dans un lieu public

Smartphone/Tablette

Autre

1. **Avez-vous déjà consulté internet pour rechercher des informations médicales? (1 réponse)**

Oui

Non

Ne me prononce pas

1. **Aviez-vous déjà entendu parler du mot « sepsis » ? (1 réponse)**

Oui

Non

Ne me prononce pas

- **Si votre réponse est « oui », où en aviez-vous entendu parler ?**

1. **Etiez-vous déjà venue(e) dans un service de réanimation (en tant que proche ou patient)?**

**(1 réponse)**

Oui

Non

Ne me prononce pas

***Depuis que votre proche est hospitalisé(e) dans le service de réanimation***

1. **Avez-vous déjà consulté des sites internet pour obtenir des informations sur les symptômes, et/ou la maladie de votre proche hospitalisé dans le service de réanimation ?** **(1 réponse)**

Oui

Non

- **Si votre réponse est « oui » (vous avez déjà consulté des sites internet pour rechercher des informations médicales), quelles sont parmi les suivantes, les deux principales raisons pour lesquelles vous êtes allé rechercher des informations médicales sur internet ? (2 réponses)**

Vous n’aviez jamais entendu parler de la maladie dont souffre votre proche

En savoir plus sur la maladie, les symptômes, qui concernent votre proche

En savoir plus sur le traitement

En savoir plus sur les médecins ou le service de réanimation qui s’occupent de votre proche

Lire des témoignages de personnes ayant eu les mêmes symptômes, maladie ou diagnostic

Etre capable de poser des questions précises au médecin avant d’aller le voir

Vérifier l’exactitude des informations médicales données par l’équipe soignante

Autre

- **Si votre réponse est « non » (vous n’avez jamais consulté des sites internet pour rechercher des informations médicales), quelles sont parmi les suivantes, les 2 principales raisons pour lesquelles vous n’êtes pas allé rechercher des informations médicales sur internet? (2 réponses)**

Absence d’accès à internet

N’utilise pas internet pour la recherche d’informations médicales

Satisfaction des informations obtenues

Absence de connaissances sur la possibilité d’obtention de telles informations sur internet

Absence de confiance sur la qualité des informations présentes sur internet

Autre

1. **Etes-vous allé directement consulter un/des site(s) internet précis ? (1 réponse)**

Oui………………Si oui, le(s)quel(s) ?

Non

Ne me prononce pas

1. **Quel moteur de recherche avez-vous utilisé? (1 réponse)**

Google

Yahoo

Autre :

1. **Avez- vous consulté le ou les sites suivants? (une ou plusieurs réponses)**

[www.wikipedia.org](http://www.wikipedia.org)

[www.doctissimo.com](http://www.doctissimo.com)

[www.vulgaris-medical.com](http://www.vulgaris-medical.com)

[www.santepratique.fr](http://www.santepratique.fr)

[www.e-sante.fr](http://www.e-sante.fr)

[www.aufeminin.com](http://www.aufeminin.com)

Dictionnaire médical en ligne

Site de la Société de Réanimation de Langue Française

Site de la Société Française d’Anesthésie-Réanimation

Sites de facultés de médecine

Sites d’associations de malades

Sites institutionnels (ex : ANSM, HAS)

Sites médicaux en langue étrangère

Site internet du service de réanimation

Forum de discussions

Autre

1. **De manière générale, les informations médicales que vous avez pu lire sur les sites internet vous paraissent-elles… ?** **(1 réponse)**

- **Fiables ?**

Oui tout à fait

Oui plutôt

Non plutôt pas

Non pas du tout

Ne me prononce pas

- **Adaptées à votre demande?**

Oui tout à fait

Oui plutôt

Non plutôt pas

Non pas du tout

Ne me prononce pas

- **Concordantes avec l’information médicale reçue dans le service ?**

Oui tout à fait

Oui plutôt

Non plutôt pas

Non pas du tout

Ne me prononce pas

1. **De manière générale, comment jugez-vous la qualité des informations médicales retrouvées sur les sites internet ? (1 réponse)**

Très satisfaisante

Satisfaisante

Moyenne

Peu satisfaisante

Insatisfaisante

Ne me prononce pas

1. **Si vous avez répondu moyenne/peu satisfaisante/insatisfaisante à la question 6, quelle est la principale raison sous-jacente? (1 réponse)**

Difficultés à trier les informations obtenues

Difficultés à évaluer la fiabilité des informations obtenues

Renseignements recherchés non trouvés

Informations obtenues de qualité insatisfaisante

Ce sont toujours les pires situations qui sont décrites sur internet

Autre

1. **Avez-vous connaissance de la procédure mise en place par la Haute Autorité de Santé (HAS) de certification des sites santé français avec la fondation « Health on the Net » ? (1 réponse)**

Oui

Non

Ne me prononce pas

1. **Avez-vous dit aux médecins s’occupant de votre proche que vous avez consulté des sites d’information médicale ou de santé sur internet ? (1 réponse)**

Oui

Non

Ne me prononce pas

- **Si votre réponse est « non », pourquoi ne dites-vous pas aux médecins de réanimation que vous consultez des sites d’information médicale ou de santé sur internet ?**

Vous faites ces recherches par curiosité et ne jugez pas nécessaire de leur en parler

Vous souhaitez comparer leur diagnostic/pronostic avec les informations que vous trouvez sur internet

Vous craignez leur réaction

Autre

1. **Auriez-vous souhaité avoir une liste de sites internet de référence (sur la maladie dont souffre votre proche) ? (1 réponse)**

Oui

Non

Ne me prononce pas

***Concernant l’information reçue dans le service de réanimation***

1. **Avez-vous reçu un livret d’accueil à la suite de votre première venue dans le service de réanimation ? (1 réponse)**

Oui

Non

Ne me prononce pas

- **Si votre réponse est « oui », que pensez-vous des informations contenues dans ce livret? (1 réponse)**

Excellent

Très bien

Bien

Mauvais

Ne me prononce pas

1. **A quelle fréquence les médecins vous ont-ils informé de l’état de votre proche ? (1 réponse)**

Très souvent

Souvent

Parfois

Rarement

Jamais

Ne me prononce pas

1. **Que pensez-vous du degré de disponibilité du personnel de réanimation pour répondre à vos questions ? (1 réponse)**

Excellent

Très bien

Bien

Mauvais

Ne me prononce pas

1. **Dans quelle mesure le personnel vous a-t-il fourni des explications compréhensibles ?**

**(1 réponse)**

Excellent

Très bien

Bien

Mauvais

Ne me prononce pas

1. **Que pensez-vous de la sincérité de l’information fournie concernant l’état de votre proche ? (1 réponse)**

Excellent

Très bien

Bien

Mauvais

Ne me prononce pas

1. **Dans quelle mesure le personnel de réanimation, vous a-t-il fourni des informations complètes (à la fois sur la maladie de votre proche et sur la prise en charge médicale)? (1 réponse)**

Excellent

Très bien

Bien

Mauvais

Ne me prononce pas

1. Que pensez-vous de la concordance des informations fournies concernant l’état de votre proche selon vos interlocuteurs locaux (Avez-vous reçu des versions similaires du médecin, personnel infirmier, etc.) (1 réponse)

Excellent

Très bien

Bien

Mauvais

Ne me prononce pas

***Concernant votre état d’esprit actuel***

1. **Vous vous sentez tendu ou énervé (1 réponse)**

Jamais

De temps en temps

Souvent

La plupart du temps

1. **Vous avez une sensation de peur comme si quelque chose d'horrible allait vous arriver**

**(1 réponse)**

Pas du tout

Un peu mais cela ne m’inquiète pas

Oui, mais ce n’est pas trop grave

Oui, très nettement

1. **Vous vous faites du souci (1 réponse)**

Très occasionnellement

Occasionnellement

Assez souvent

Très souvent

1. **Vous pouvez rester tranquillement assis à ne rien faire et vous sentir décontracté**

**(1 réponse)**

Oui, quoi qu’il arrive

Oui, en général

Rarement

Jamais

1. **Vous éprouvez des sensations de peur et avez l'estomac noué (1 réponse)**

Jamais

Parfois

Assez souvent

Très souvent

1. **Vous avez la bougeotte et n'arrivez pas à tenir en place (1 réponse)**

Pas du tout

Pas tellement

Un peu

Oui, c’est tout à fait le cas

1. **Vous éprouvez des sensations soudaines de panique (1 réponse)**

Jamais

Pas très souvent

Assez souvent

Vraiment très souvent

1. **Vous prenez plaisir aux mêmes choses qu'autrefois (1 réponse)**

Oui, tout autant

Pas autant

Un peu seulement

Presque plus

1. **Vous riez facilement et voyez le bon côté des choses (1 réponse)**

Autant que par le passé

Plus autant qu'avant

Vraiment moins qu'avant

Plus du tout

1. **Vous êtes de bonne humeur (1 réponse)**

La plupart du temps

Assez souvent

Rarement

Jamais

1. **Vous avez l'impression de fonctionner au ralenti (1 réponse)**

Jamais

Parfois

Très souvent

Presque toujours

1. **Vous ne vous intéressez plus à votre apparence (1 réponse)**

Vous y prêtez autant d'attention que par le passé

Il se peut que vous n’y fassiez plus autant attention

Vous n'y accordez pas autant d'attention que vous devriez

Plus du tout

1. **Vous vous réjouissez d'avance à l'idée de faire certaines choses (1 réponse)**

Autant qu'avant

Un peu moins qu'avant

Bien moins qu'avant

Presque jamais

1. **Vous pouvez prendre plaisir à un bon livre ou à une bonne émission radio ou télévision**

**(1 réponse)**

Souvent

Parfois

Rarement

Très rarement

***Vous êtes…***

1. **Une femme**

**Un homme**

1. **Votre âge :**
2. **Votre relation avec la personne hospitalisée en réanimation**

Epouse  Mère  Sœur  Fille  Conjoint(e)

Epoux  Père  Frère  Fils  Autre, précisez :

1. **Votre niveau d’études:**

Sans diplôme

Brevet des collèges

CAP/BEP (autres diplômes techniques)

Baccalauréat (général, pro et technologique)

Bac+2 (BTS ou autre)

Bac+3/4 (Licence, Maîtrise)

Bac+5 (Master, écoles d'ingénieur, écoles d'arts...)

Bac+7 etc (Doctorat, post-doc, thèse)

1. **De manière générale, quel est votre degré de satisfaction sue la prise en charge actuelle de votre proche dans le service de réanimation ? (1 réponse)**

Très satisfait

Satisfait

Peu satisfait

Pas satisfait

Ne se prononce pas

***Nous vous remercions vivement pour votre participation à cette étude ☺***

***Nous espérons que les données recueillies nous permettront de mieux répondre aux attentes des proches hospitalisés dans les services de réanimation.***

***Cordialement,***

***Dr. YL Nguyen et Pr. JP Mira***

**Statistical analyses**

Associations of ICU, patient and responder's characteristics with Internet use were analyzed using logistic regression models (three-stage procedure). First, basic characteristics of the responder were analyzed in a multivariable model to determine a set of potential confounders. Significant variables in these analyses were used systematically as adjustment factors in subsequent analyses. Then a potential clustering effect on the center was investigated using a mixed effects logistic regression model, with a random center effect. The standard deviation of the center effect relative to the effect of adjustment variables was taken as a measure of clustering, and tested using a permutation test (12). In case of a significant or a substantial center effect, subsequent analyses where adjusted on the center using random mixed-effects logistic regression models; otherwise, logistic regression models with only mixed effects and without center were used. In the third step, other variables related to the ICU, the patient or the responder where analyzed.

Analyses were performed using the R statistical software version 3.0.2 (13).
