# Supplementary material for: “RéaNet”, the Internet utilization among surrogates of critically ill patients with sepsis
Source: PLoS One. 2017 Mar 30;12(3):e0174292. doi: 10.1371/journal.pone.0174292 (PMC5373530; doi:10.1371/journal.pone.0174292)
Supplement: S1 Table — (DOCX) [file pone.0174292.s003.docx]

**Table 1: ICUs characteristics (N=19)**

| Parameters | N or median or mean | (%) or (quartiles) or (standard deviation) |
| --- | --- | --- |
| University hospitals | 17 | (89) |
| ICU beds | 20 | (20-24) |
| Post-ICU beds | 16 | (14-20) |
| Senior physicians | 8 | (7-9) |
| Junior physicians | 6 | (4-8) |
| Nurse-to-patients ratio | 0.36 | 0.05 |
| ICU web site | 3 | 16 |
| Consultations post ICU stay | 6 | 32 |
| Annual total volume | 900 | (660-1164) |
| Annual sepsis volume | 100 | (100-142) |
| ICU mortality | 21 | 7 |
