# Supplementary material for: “RéaNet”, the Internet utilization among surrogates of critically ill patients with sepsis
Source: PLoS One. 2017 Mar 30;12(3):e0174292. doi: 10.1371/journal.pone.0174292 (PMC5373530; doi:10.1371/journal.pone.0174292)
Supplement: S2 Table — (DOCX) [file pone.0174292.s004.docx]

**Table 2: Centers organization on medical information (N=19)**

| Characteristics | N | % |
| --- | --- | --- |
| Internet access in the ICU for patients and families | 0 | 0 |
| Waiting room available | 19 | (100) |
| Room used only for family information available | 17 | (89) |
| Family information leaflet available | 18 | (95) |
| Social worker | 17 | (89) |
| Psychologist | 10 | (53) |
| 24/7 visiting hours | 6 | (32) |
| Written protocol for interacting with families | 2 | (11) |
| Family information delivery by junior physicians   - often - sometimes - very occasionally - not at all | 1  1  14  3 | (5)  (5)  (74)  (16) |
| Participation of nursing staff during family information meetings   - often - sometimes - very occasionally - not at all | 5  9  5  0 | (26)  (47)  (26)  (0) |
