# Supplementary material for: “RéaNet”, the Internet utilization among surrogates of critically ill patients with sepsis
Source: PLoS One. 2017 Mar 30;12(3):e0174292. doi: 10.1371/journal.pone.0174292 (PMC5373530; doi:10.1371/journal.pone.0174292)
Supplement: S3 Table — (DOCX) [file pone.0174292.s005.docx]

**Table 3: Patients characteristics (N=146)**

| Parameters | N or mean or median | (%) or (standard deviation) or(quartiles) |
| --- | --- | --- |
| Age | 64 | (16) |
| Male | 100 | (68) |
| MacCabe   - Category1 (non fatal) - Category 2 (ultimately fatal disease) - Category 3 (rapidly fatal disease) | 75  47  24 | (51)  (32)  (16) |
| SAPS2 score | 53 | (17) |
| Sepsis origin   - pulmonary - gastro-intestinal - urinary - cutaneous - other | 70  25  16  13  22 | (48)  (17)  (11)  (9)  (15) |
| Organ supplementation   - vasopressors - invasive mechanical ventilation - non-invasive mechanical ventilation - renal replacement therapy | 117  116  26  36 | (83)  (82)  (18)  (26) |
| Decision to withdrawal/withholding life sustaining therapies | 30 | (21) |
| ICU mortality | 35 | (24) |
| ICU length of stay | 11 | (7-18) |
