# Supplementary material for: “RéaNet”, the Internet utilization among surrogates of critically ill patients with sepsis
Source: PLoS One. 2017 Mar 30;12(3):e0174292. doi: 10.1371/journal.pone.0174292 (PMC5373530; doi:10.1371/journal.pone.0174292)
Supplement: S4 Table — (DOCX) [file pone.0174292.s006.docx]

**S4 Table : Internet use characteristics (N=169)**

| Parameters | N | (%) |
| --- | --- | --- |
| Internet use for general purpose | 139 | (83) |
| Internet use for seeking health information online | 115 | (83) |
| Internet use for seeking health information on their proxy | 77 | (55) |
| Internet connection:   - Home - Smartphone - Work - Public place | 125  49  26  3 | (79)  (31)  (16)  (2) |
| Knowledge of the web sites certification “Health on the net” | 12 | (8) |
| Willingness to obtain a list of websites on sepsis | 76 | (55) |
