# Supplementary material for: “RéaNet”, the Internet utilization among surrogates of critically ill patients with sepsis
Source: PLoS One. 2017 Mar 30;12(3):e0174292. doi: 10.1371/journal.pone.0174292 (PMC5373530; doi:10.1371/journal.pone.0174292)
Supplement: S5 Table — (DOCX) [file pone.0174292.s007.docx]

**S5 Table: Internet users characteristics (N=77)**

| Parameters | N | % |
| --- | --- | --- |
| Internet use motivations:   - Learn more about sepsis - Learn more about treatments - Be able to ask questions to physicians - Never heard about sepsis - Read testimonials - Verify information given - Learn more about ICU physicians or the ICU | 65  26  25  18  11  2  1 | (84)  (34)  (32)  (23)  (14)  (3)  (1) |
| Who informed the doctors they used internet | 24 | (32) |
| Reasons Internet users did not inform the doctors about internet use:   - Curiosity and no-necessity to share it with doctors - Willingness to do comparisons - Fear doctor reaction - Other | 36  5  2  7 | (72)  (10)  (4)  (14) |
| Consultation of a specific website as first line | 15 | (21) |
| Websites most-frequently used:   - www.fr.wikipedia.org - www.doctissimo.com - [www.e-sante.fr](http://www.e-sante.fr) - www.vulgaris-medical.com - www.sante-pratique.fr - Medical dictionary online - Discussion forums - [www.aufeminin.com](http://www.aufeminin.com) - French societies of critical care websites - Medical schools websites - Patients associations websites - National institutions websites - Foreign health information websites - Others | 52  45  21  11  11  11  11  10  8  7  4  3  3  3 | (69)  (60)  (28)  (15)  (15)  (15)  (15)  (13)  (11)  (9)  (5)  (4)  (4)  (4) |
| They found reliable information on the internet:   - Strongly agree - Agree - Disagree - Strongly disagree - Undecided | 7  52  1  0  15 | (9)  (69)  (1)  (0)  (20) |
| They found appropriate information on the internet:   - Strongly agree - Agree - Disagree - Strongly disagree - Undecided | 10  45  10  1  9 | (13)  (60)  (13)  (1)  (12) |
| They found concordant information on the internet:   - Strongly agree - Agree - Disagree - Strongly disagree - Undecided | 15  46  1  1  11 | (20)  (62)  (1)  (1)  (15) |
| They assess quality information on the internet:   - Very good - Good - Barely acceptable - Poor - Very poor - Undecided | 2  37  27  4  0  4 | (3)  (50)  (36)  (5)  (0)  (5) |
