# Supplementary material for: “RéaNet”, the Internet utilization among surrogates of critically ill patients with sepsis
Source: PLoS One. 2017 Mar 30;12(3):e0174292. doi: 10.1371/journal.pone.0174292 (PMC5373530; doi:10.1371/journal.pone.0174292)
Supplement: S6 Table — (DOCX) [file pone.0174292.s008.docx]

**S6 Table: Potential confounders of internet utilization**

| Variable | OR (95% CI) | p |
| --- | --- | --- |
| Responder age (/10yrs) | 0.90 [0.68; 1.20] | 0.48 |
| Male responder | 1.36 [0.55; 3.35] | 0.51 |
| Education: high school | 1.30 [0.33; 5.04] | 0.71 |
| Education: college | 1.35 [0.33; 5.47] | 0.67 |
| Education: graduated college | 0.68 [0.23; 1.96] | 0.47 |
| Prior health-related internet utilization | 20.7 [4.30; 100.1] | 0.0002 |
